# Point-of-care testing in the diagnosis of infectious diseases

**DOI:** 10.3389/fmicb.2026.1949318

**Published:** 2026-10-19

**Authors:** Quan Fang, Yuyan Lin, Guiqi Hu, Jiangjie Hu, Jun Cheng, Yuzhu Dai, Li Yan, Xiaohong Wu

**Affiliations:** 1 Department of Clinical Laboratory, Air Force Hangzhou Special Service Convalescent Center Convalescent Hospital Area 1, Hangzhou, Zhejiang, China; 2 Department of Clinical Laboratory, Second People's Hospital, Lishui, Zhejiang, China; 3 School of Laboratory Medicine, Bengbu Medical University, Bengbu, Anhui, China; 4 Department of Clinical Laboratory, The 903rd Hospital of The People's Liberation Army, Hangzhou, Zhejiang, China; 5 Department of Health Economic, The 903rd Hospital of The People's Liberation Army, Hangzhou, Zhejiang, China

**Keywords:** isothermal amplification, lateral flow assay, microfluidic chip, point-of-care testing, rapid diagnosis, sample to answer

## Abstract

Point-of-care testing (POCT) has emerged as an indispensable diagnostic strategy for addressing the increasing global burden of infectious diseases and the worsening threat of antimicrobial resistance. Owing to prominent advantages such as rapid detection of infectious agents, portability, and ease of accessibility, POCT can effectively address the inherent limitations of traditional laboratory diagnostic workflows. This review summarizes the core technical platforms that underlie current POCT technologies, including lateral flow assays, chemiluminescence immunoassays, isothermal amplification systems, CRISPR/Cas-based diagnostic systems, and microfluidic chip technologies. Furthermore, we describe the key clinical scenarios in which POCT can be applied, including respiratory tract, bloodstream, gastrointestinal, urinary tract, and sexually transmitted infections. We also comprehensively discuss the technical characteristics and clinical applicability of mainstream POCT modalities, including immunochromatography and molecular diagnostics. Despite limitations associated with detection sensitivity, quality control, cost efficiency, and clinical integration, POCT technology continues to undergo consistent improvements. Advances in nanomaterials, microfluidic engineering, and artificial intelligence and continuous improvements in industry standardization have led to increased performance, systematic integration, and intelligent detection in POCT. Given these technological advancements and optimizations, POCT shows promising applicability and is therefore expected to become a core diagnostic tool for both precision clinical management and epidemic prevention and control in public health.

## 1 Introduction

The incidence of infectious diseases has been increasing worldwide, placing ever greater pressure on public health. According to the Global Burden of Disease study, infectious diseases remain a leading cause of death despite the latest advancements in medical care (Peng et al., 2026). In early 2026, infectious diseases that required close attention in China included influenza, norovirus, Mpox, avian influenza, and several seasonal illnesses (Huang et al., 2026). An additional concern is the worsening problem of antimicrobial resistance (AMR), which is driven by excessive antibiotic overuse. For instance, data from a tertiary hospital in China revealed that the rate of *Enterococcus faecium* resistance to vancomycin increased from 1.6% in 2019 to 23.1% in the first two quarters of 2025 (Li et al., 2026c). This tendency not only makes common infections more difficult to treat but also fuels the spread of resistant bacteria across humans, animals, and the environment.

Traditionally, infectious diseases are diagnosed with large, centralized laboratory equipment (Table 1). For respiratory infections, laboratories often use large multiplex PCR platforms such as the Roche Cobas 6800/8800 (Matic et al., 2024), whereas bloodstream infections (BSIs) require a full suite of instruments, including the BD BACTE blood culture system, the VITEK MS mass spectrometer, and the VITEK 2 system for susceptibility testing (Church et al., 2025), resulting in a 2–4-day turnaround to obtain the results. For gastrointestinal infections, stool samples are incubated in a CO₂ incubator for 24–72 h before they are subjected to mass spectrometry. The assessments of sexually transmitted infections (STIs) usually start with screening using an automated chemiluminescence analyzer, followed by confirmation through Western blotting. The relevant instruments are bulky (often occupying one to two square meters), expensive (costing hundreds of thousands to more than a million yuan per unit) and complex (requiring trained technicians). Consequently, clinicians often have to rely on empirical prescriptions during the critical window for treatment, which may lead to suboptimal antibiotic selection. One study reported that approximately 34% of patients with sepsis received inappropriate empirical antibiotic therapy (Shappell et al., 2025), whereas in a study on patients with urinary tract infections, those whose empirical treatments matched the susceptibility results tended to recover faster (Ng et al., 2025).

**Table 1:** Application of traditional diagnostic techniques in infectious diseases.

**Infection type**	**Traditional lab equipment**	**Equipment features**	**Process**	**Turnaround time**
Respiratory tract infections	Fully automatic high-throughput molecular diagnostic instrument (Roche cobas 6800/8800) (Matic et al., 2024)	Large, professional lab	Sample → central lab → Multiplex PCR → Results returned	1–5 h
Bloodstream infections	Blood culture system (BD BACTEC™), Mass spectrometer (VITEK MS) (Church et al., 2025), Biochemical identification and drug sensitivity device (VITEK 2) (Otto-Karg et al., 2009)	Multiple devices, high costs, large	Blood collection → Blood culture → Mass spectrometry identification → Drug sensitivity analysis (Wu et al., 2026)	2–4 days
Gastrointestinal infections	CO₂ incubator, Mass spectrometer (VITEK MS), molecular diagnostic instrument (QuantStudio PCR), ELISA (Tecan immunoanalyzer) (Lopez Muñoz et al., 2024)	Multiple devices, require biosafety cabinet, incubators	Stool processing → Culture (24–72 h) → Mass spectrometry/molecular identification	2–5 days
Urinary tract infections	Fully automatic urine analyzer (Sysmex UF-5000) (ClinicalTrials.gov, 2025), Biochemical identification and drug sensitivity device (VITEK2) (Otto-Karg et al., 2009)	Urine analyzer + susceptibility system	Urine screening → Culture → Susceptibility testing	24–72 h
Sexually transmitted infections	Microscopy and culture system, molecular diagnostic instrument, NAATs (BD Max) (Caruso et al., 2021)	Large, high costs	Preliminary serological screening → Confirmation of positive samples	2–3 days

NAAT, nucleic acid amplification test; ELISA, enzyme-linked immunosorbent assay; PCR, polymerase chain reaction; CO₂, carbon dioxide; BD BACTEC, Becton Dickinson BACTEC blood culture system; VITEK MS, VITEK mass spectrometer; VITEK 2, VITEK 2 automated microbiology system.

In the current healthcare landscape, the need for fast and accurate diagnostic tools has become more urgent than ever to address public and individual health demands. In terms of public health, rapid diagnostics can detect emerging pathogens in real time, track resistance trends, and limit the hospital spread of infections. At the individual level, traditional centralized testing has clear limitations such as long operation times, high operational demands, and significant equipment costs. To solve these issues, point-of-care testing (POCT), defined as the medical diagnostic testing of patients at or near the site of patient care—including testing with compact devices in the emergency room, outpatient clinic, or primary care setting or with a self-test kit at home, has been proposed. The results of these tests may lead to changes in clinical decision-making (Larkins et al., 2026). Point-of-care tests can be broadly characterized into five primary groups. The first group covers rapid immunodiagnostics using lateral flow test strips, which remain the most mature and ubiquitously adopted POCT format. The second group consists of compact chemiluminescence immunoassays that support quantitative readouts, balancing strong analytical sensitivity and reliable quantification. The third group includes isothermal nucleic acid amplification methods such as loop-mediated isothermal amplification (LAMP) and recombinase polymerase amplification (RPA), which permit highly sensitive nucleic acid testing using portable formats. The fourth group includes CRISPR/Cas-based next-generation molecular diagnostic methods, which have outstanding target specificity. The final group consists of microfluidic chip systems, which embody the evolving paradigm of fully automated, seamless sample-to-answer testing. The core principle of POCT is to achieve rapid testing close to the patient while covering a wide range of potential targets. By bringing testing from the central laboratory to the patient’s bedside with portable equipment, POCT enables a “sample-to-answer” model (Shaikh et al., 2025) that can improve efficiency in emergency departments (EDs) and intensive care units (ICUs). For example, the research-grade Lodestar DX system developed by Lusern Scientific uses isothermal amplification to identify six pathogens in only 35 min (Diggle et al., 2024). In respiratory infections, the BioFire FilmArray can test for nearly 20 respiratory pathogens and 7 genetic resistance markers in approximately 75 min using microfluidics methods (Mustafa Hellou et al., 2025). These technologies are not only fast but can also be placed at the exact point of care, thus helping clinicians make better decisions, use antibiotics more accurately, and slow the spread of AMR.

Compared with existing POCT reviews, this paper is the first to describe a multilevel analytical framework that covers topics from immunochromatography to fully integrated molecular detection and highlights the logic of technology selection under different clinical scenarios. Additionally, this review emphasizes AMR surveillance and epidemic prevention and control, incorporating resistance gene detection and phenotypic drug susceptibility testing into the discussion. Finally, implementation and standardization issues in resource-limited regions are covered, including practical factors such as cost, training, quality control and medical insurance policies, to improve the translational value of the review.

### 1.1 Literature search strategy

This narrative review was conducted according to a systematic review procedure. We searched the PubMed, Web of Science, Scopus, and China National Knowledge Infrastructure (CNKI) databases for manuscripts published from January 2016 to June 2026. The main search terms were “point-of-care testing” or “POCT” or “rapid diagnostic test” and “infectious disease” or “pathogen detection” or “antimicrobial resistance,” as well as proper nouns for the respective technical platforms (e.g., “lateral flow immunoassay”, “isothermal amplification”, “CRISPR/Cas”, and “microfluidics”). In terms of manuscript topics, we searched for studies involving the application of POCT technologies in the diagnosis of infectious diseases, which yielded original studies or systematic reviews that were published in peer-reviewed journals. Case reports, conference abstracts, and non-English and non-Chinese studies were excluded. The references of the included articles were also searched for relevant publications. This review was reported in accordance with the PRISMA guidelines, although no quantitative meta-analysis was performed. A small number of relatively old references (e.g., the original descriptions of LAMP in 2000, helicase-dependent amplification (HDA) in 2004, and RPA in 2006) are cited despite being published outside the retrieval window solely as technical milestones to provide essential background information on the development of the corresponding platforms.

## 2 Primary technological platforms that underlie POCT

### 2.1 Lateral flow immunoassay test strips

Among the POCT platforms currently in use, immunochromatography is the most mature and widely deployed technology. The representative format, lateral flow immunoassay (LFIA) test strips, have been extensively translated into real-world use across clinical diagnostics, population-level public health surveillance, and at-home self-monitoring (Park, 2022; Prakash et al., 2026). LFIA is an immunodetection method based on capillary action and specific antigen–antibody binding. A standard strip assembly consists of four core functional components: a sample pad, a conjugate pad, a nitrocellulose (NC) membrane and an absorbent pad. Upon loading of the sample onto the sample pad, capillary action drives movement of the liquid through sequential zones on the strip. Target analytes bind prelabelled antibodies within the conjugate pad to form immune complexes, which continue flowing through the strip until they are captured by immobilized antibodies printed on the NC membrane; visible color signals subsequently develop at the test line. The full assay is completed in 15 min, requires no specialized operator training, and generates results that can be read by the naked eye. LFIAs are well suited for decentralized testing and untrained self-testing because of their speed, minimal handling steps and low manufacturing costs (Ince and Sezgintürk, 2022; Sohrabi et al., 2022). For infectious disease diagnostics, mainstream commercial LFIA products include antigen test kits for SARS-CoV-2 and influenza, rapid antibody detection strips for HIV, and semiquantitative assays targeting PCT and CRP. Nevertheless, LFIAs have limited analytical sensitivity associated with the ability to label reporters and intrinsic membrane material performance, and it predominantly provides qualitative or semiquantitative results (Fang et al., 2025). Such constraints increase the risk of false-negative outcomes, particularly when samples contain low viral loads or were obtained during the seroconversion window (during which there is delayed viral antibody production).

Notably, most clinical evaluations of LFIAs adopted cross-sectional diagnostic accuracy designs, with sample sizes generally ranging from 100 to 500; some small-scale studies have even involved fewer than 50 samples (Kirby et al., 2024; Beaubien-Souligny et al., 2025). Although methods such as the Buderer formula are commonly used for sample size calculation, many studies have failed to report the rationale for their sample sizes. In terms of head-to-head comparisons, a 2026 multicenter evaluation of five monkeypox LFIA test strips revealed substantial differences in sensitivity among products from different brands (39.2–59.8%), while specificity remained above 95% for all tested products (Kavunga-Membo et al., 2026). These findings indicate that product performance differences within the same technical platform cannot be ignored and that interested parties should carefully distinguish specific products and their validation populations when reviewing LFIA performance data. In addition, most LFIA studies have demonstrated favorable performance under ideal laboratory conditions, yet data concerning operator dependency and environmental adaptability in real-world primary care and home-use settings remain limited (Tanifuji and Citterio, 2026).

### 2.2 Chemiluminescence immunoassays

Chemiluminescence immunoassays (CLIAs) combine highly sensitive chemiluminescence reactions with highly specific immune reactions and serve as a key technical transition from traditional immunochromatography to high-performance quantitative detection. CLIAs can be categorized into two main types. The first type is enzymatic chemiluminescence assays, most of which use HRP to catalyse luminol or its derivatives to produce light at 425 nm, while others use ALP to catalyse AMPPD to produce luminescence. An enhancer (e.g., potassium ferricyanide and potassium periodate) can be added to further increase electron activation, leading to increased analytic sensitivity. The second type is direct chemiluminescence assays, which typically use acridinium esters as luminophores conjugated directly to the primary monoclonal antibody to generate a signal. Acridinium esters are highly specific labels that spontaneously oxidize in the presence of alkaline hydrogen peroxide, producing a signal at 429 nm within 2 s, whereas luminol generates a longer-lasting signal (Merck, 2026). Compared with conventional immunochromatography, CLIAs are particularly suitable for detecting inflammatory markers and hormones because of their superior sensitivity and wide linear detection range. For instance, Sun et al. (2025) developed a fully automated chemiluminescence immunoassay analyzer that requires no excitation light source and enables the onsite measurement of serum AMH. Under optimal conditions, its limit of detection reaches 0.02 ng/mL, with a linear range of 0.02–25 ng/mL. However, CLIA instruments are relatively bulky (most are bench-top devices), and the cost per individual test is higher than that of LFIA test strips, limiting their use in community and at-home self-testing settings.

Clinical validation studies for CLIA platforms tend to use standardized designs, with some studies adopting multicenter prospective designs and sample sizes ranging from over one hundred to several hundred cases. Notably, however, among the currently published CLIA-POCT studies, head-to-head comparisons with centralized CLIA platforms remain limited (Younes et al., 2026). Most studies have compared CLIA-POCT with only ELISAs or traditional immunochromatography methods and lack parallel validation against large-scale instruments on the same platform. In terms of costs, despite their miniaturization, the reagent cost per CLIA and instrument maintenance expenses are still markedly higher than those of LFIAs, which creates practical barriers to their use in grassroots and community-based settings. CLIA platforms also have higher requirements for equipment calibration and quality control materials, placing greater demands on the operating environment and operator proficiency.

### 2.3 Isothermal amplification technology

Isothermal amplification technology (IAT) has become the gold standard for laboratory diagnostics and POCT in molecular diagnostics, representing a crucial shift from immunological assays to nucleic acid detection. IAT uses an enzyme with strand displacement activity that can unwind DNA double strands and synthesize new strands under constant temperature to exponentially amplify nucleic acids. Compared with traditional PCR, IAT does not require complex temperature cycling for denaturation, annealing or extension; the reliance on sophisticated temperature control modules is reduced, greatly improving portability in POCT applications to monitor sudden infectious disease outbreaks (Zhao et al., 2015; Bi et al., 2025; Xie et al., 2026). IAT can be divided into different types according to the underlying technological principles. In 1991, Compton (1991) first reported nucleic acid sequence-based amplification (NASBA), pioneering the application of IAT in RNA detection. In 2000, Notomi et al. (2000) achieved another great breakthrough in Japan with the development of LAMP, which quickly became one of the most widely used IATs owing to its high specificity. Afterwards, the field of IAT entered rapid development. In 2004, Vincent et al. (2004) developed HDA. In 2006, Piepenburg’s team developed RPA, which requires a relatively low reaction temperature (37 to 42 °C) (Piepenburg et al., 2006). These technologies hold great potential for scenarios such as rapid diagnosis and onsite testing. However, the limitations of IAT are obvious and include the relatively complicated design of primers and the tendency for the risk of nonspecific amplification to increase with the use of multiple primers.

Considerable heterogeneity exists in the designs of clinical validation studies for IATs. For tuberculosis diagnosis, an RT-LAMP validation study published in 2025 rigorously applied the Buderer formula to estimate a sample size of 350 cases, adopted both MGIT culture and Xpert MTB/RIF as reference methods, and reported relatively reliable sensitivity and specificity data (Nair et al., 2025). Nevertheless, many isothermal amplification studies have remained at the laboratory optimization stage, and clinical sample validation with fewer than 100 cases is common among other studies. With respect to SARS-CoV-2, one study compared the RHAM isothermal-amplification-based Pluslife assay with the EasyNAT assay and Wondfo antigen detection test strips and showed that both molecular detection platforms achieved a positive concordance rate of 96.30%, significantly higher than the 81.48% of the antigen test strips (Zhong et al., 2025). Such head-to-head data serve as a valuable reference for clinical selection, but similar studies remain relatively scarce. Particularly, almost no studies have directly compared multiplex assays and different IATs (e.g., LAMP vs. RPA vs. NASBA).

### 2.4 CRISPR/Cas

The CRISPR/Cas system has recently emerged as one of the most extensively studied technologies in the field of molecular diagnostics (Zhang et al., 2022). Unlike conventional nucleic acid detection methods, CRISPR-based diagnostics capitalize on the “trans-cleavage” activity of Cas proteins upon its recognition of target nucleic acids. These proteins nonspecifically cleave adjacent single-stranded reporter molecules to generate detectable signals (Lau et al., 2025). The most widely applied proteins in the CRISPR/Cas system are Cas12 and Cas13 (Chen et al., 2023; Lau et al., 2025). Cas12 targets double- or single-stranded DNA, while Cas13 targets RNA; both proteins exhibit both cis-cleavage and trans-cleavage activities. Currently, there are two primary strategies for implementing CRISPR diagnostics (Yi B. et al., 2025). In the first, Cas proteins recognize a target that is preenriched via isothermal amplification (e.g., RPA or LAMP) and trigger trans-cleavage to produce signals (Zhang et al., 2022; Yi B. et al., 2025). This alternative approach enables direct detection without preamplification, resulting in a simpler and faster technique that is more amenable to integration into portable devices (Yi B. et al., 2025). In the detection of infectious diseases, SHERLOCK and DETECTR represent two well-established platforms (Mustafa and Makhawi, 2021; Mayuri et al., 2026). SHERLOCK, which is based on Cas13a, is employed for detecting RNA viruses, whereas DETECTR, which relies on Cas12a, is used for DNA virus detection. CRISPR diagnostics are among the key methods for HPV testing (He et al., 2025), and CRISPR/Cas has also been used for the detection of pathogens such as SARS-CoV-2, monkeypox, dengue virus, and *Mycobacterium tuberculosis*.

Unfortunately, prominent evidence gaps exist in terms of the efficacy of CRISPR-based diagnostics. Although encouraging analytical sensitivity (down to the aM level) has been reported at the laboratory scale, clinical validation generally lacks sufficient scale and rigor. Most reported studies on CRISPR-based diagnostics are proof-of-concept investigations or retrospective analyses with small sample sizes, which often range from 50 to 150 samples, while some studies have even included fewer than 30 samples. A 2025 review explicitly stated that CRISPR diagnostics must evolve beyond technical optimization to embrace ecological adaptability (Wang et al., 2025b). Specifically, the major barriers to clinical translation include interference from inhibitors in complex samples, limitations on large-scale manufacturing, the lack of multicenter clinical data, and different regulatory requirements across regions. Research on the detection of ESKAPE multidrug-resistant bacteria remains fragmented, and the essential trade-offs among sensitivity, multiplex detection performance and ease of use have not been adequately addressed (Qian et al., 2023). In addition, ongoing controversies and concerns persist regarding the quantitative capability of CRISPR-based strategies. CRISPR/Cas performs well qualitatively, but accurate, quantitative determination of the pathogen load remains challenging (Yao et al., 2025). Moreover, among approved POCT platforms, only a small number of products based on CRISPR technology have obtained approval from major regulatory authorities; the vast majority of platforms remain suitable for use only in research (Zhou et al., 2025).

### 2.5 Microfluidic technology

Microfluidic technology can be described as the miniaturization and integration of conventional laboratory workflows, including sample lysis, nucleic acid extraction, target amplification, and signal detection, onto a single and self-contained platform, typically a chip measuring only a few centimeters in size. Upon introduction of an unprocessed sample, the system automatically executes the full analytical cascade (Yuan et al., 2025; Ou et al., 2026). Within the chip, fluid actuation is frequently achieved through passive mechanisms, most notably capillary action, which further allows the size of the chip to be minimized. Other miniaturization methods include the use of filter paper instead of traditional silicon or glass chips (Soum et al., 2019; Li et al., 2020; Asci Erkocyigit et al., 2023). In contrast, centrifugal microfluidics, as embedded in platforms such as the “lab-on-a-CD,” leverage rotational forces for precisely controlling liquid handling, thereby improving robustness and usability in low-resource and point-of-care settings (Peshin et al., 2022; Xiao et al., 2022; Yuan et al., 2025). In recent years, more research teams have advanced the engineering of multiscale hierarchical architectures on paper, such as ordered micropillar arrays and colloidal crystal templates, to enable pump-free fluid transport and improve optical readouts of fluorescence and chemiluminescence (Gao et al., 2019). Furthermore, the incorporation of nanozyme catalysts into paper-based microfluidic assays has significantly improved detection performance (Kishnani et al., 2021; Li et al., 2024). Acoustofluidics, another emerging frontier, synergistically combines acoustic wave manipulation with microfluidic confinement to achieve on-chip particle separation, cell enrichment, and integrated sample-to-result analysis (Chen et al., 2026). Collectively, the key strengths of microfluidics are its functional integration and process automation capabilities, which lower the demands on operators and mitigate cross-contamination risk (Li et al., 2026a).

Nevertheless, the scalability of microfluidic chips is complex because of challenges associated with fabrication reproducibility, material compatibility, and manufacturing costs, which impede broad deployment in resource-constrained environments. A 2026 review analyzing nearly 100 reports on microfluidic paper-based analytical devices (μPADs) revealed that despite the number of laboratory-scale proof-of-concept studies, very few involved validation with authentic clinical samples while delivering “sample-to-answer” performance (Tanifuji and Citterio, 2026). Clinical validation studies were typically performed with limited sample sizes, ranging from 25 to 140 subjects (García-Azuma et al., 2024). Key translational bottlenecks include complex clinical sample processing, chip multifunction integration, manufacturing reproducibility for large-scale production, and ambiguous regulatory approval pathways. Although certain sub-disciplines, such as centrifugal microfluidics, have attained relatively high technological readiness, they still face engineering barriers related to fabrication consistency, valve performance, and hydrophilic-coating stability (Peshin et al., 2022; Wang et al., 2026). Notably, several microfluidic platforms (e.g., BioFire FilmArray and Cepheid GeneXpert) have been successfully commercialized and have secured regulatory authorization. Nonetheless, such successful implementations are largely limited to some large enterprises, while numerous microfluidic innovations originating from academic research fail to pass through the product-translation pipeline.

## 3 POCT in infectious diseases

### 3.1 Respiratory infections

The cospread of multiple respiratory viruses poses significant challenges for rapid triage and precision treatment in outpatient clinics (Ramirez-Priego et al., 2026). Immunochromatography is the most widely used and well-established technology for viral antigen POCT (Sohrabi et al., 2022) and is commonly used for the combined screening of COVID-19 and influenza among respiratory infections (Yamagishi et al., 2024; Cohen et al., 2025). By 2026, several companies have launched home-use antigen tests for influenza A/B, respiratory syncytial virus (RSV), and adenovirus, offering both professional and self-test options and meeting public demand for quick self-assessment. In October 2024, the Healgen combined rapid antigen test for COVID-19, influenza A, and influenza B, developed by Orient Gene and classified as *De Novo* by the FDA, became the first over-the-counter (OTC), home-use triple test in the U.S. that delivered results in approximately 15 min (ABC NEWS, 2024; FDA, 2024). Recently, researchers at Tianjin Medical University developed a double-drive microfluidic chip system that simultaneously detects RSV, influenza A/B, and SARS-CoV-2 in only 5 min using only a 35 μL sample (Yang et al., 2026). Nevertheless, immunochromatography has clear limitations. Most of these tests provide qualitative or semiqualitative results, and the sensitivity tends to be relatively low due to issues with labelling materials and membranes; thus, false negatives can occur when viral loads are low or antibodies have not yet been produced (Bayart et al., 2023; Murphy et al., 2024; Savolainen et al., 2025; Yang et al., 2026).

With respect to rapid nucleic acid testing, the integration of PCR with microfluidic chips has greatly improved the portability of POCT while becoming a hot topic in molecular diagnostics (Zhao et al., 2015; Bi et al., 2025; Xie et al., 2026). The prototype Coyote Bio Flash10 platform uses rapid heating and cooling for achieving nucleic acid extraction, amplification, and detection in 30 to 36 min. In the detection of SARS-CoV-2, influenza A/B, and RSV, the overall agreement rate of this prototype with the industry gold standard (GeneXpert) was 94.6% (Smit et al., 2025). Zheng et al. (2021) developed a portable POCT tool that combines LFIAs to detect the SARS-CoV-2 nucleic acid sequence. Their RT-RAA/LFD method achieved a detection limit of 1 copy/μL with no cross-reactivity and showed 100% sensitivity and specificity relative to RT–qPCR in 100 clinical samples (Zheng et al., 2021). Luo et al. (2026) developed a G4-NASBA detection system on the basis of NASBA that can detect influenza A virus RNA at concentrations as low as 3 copies/μL in 15 min without cross-reactivity from other viral RNAs. With regard to multiplex detection, BioFire SPOTFIRE can identify nearly 20 respiratory pathogens in approximately 15 min (Meltzer et al., 2026). Patricia and colleagues developed a microfluidic biosensor using surface plasmonic resonance that can simultaneously quantify SARS-CoV-2, influenza A/B, and RSV antigens in a single test. The detection limit of this biosensor was within the ng/mL range, while clinical validation showed 97.22% sensitivity and 100% specificity relative to RT–PCR among 108 clinical samples (Ramirez-Priego et al., 2026).

### 3.2 Bloodstream infections

BSIs caused by pathogens can lead to systemic symptoms, and for every hour of treatment delay, the survival rate decreases by up to 7.6% (Wu et al., 2026). Circulating inflammatory markers in the blood play key roles in the early identification and dynamic monitoring of BSIs. Clinical POCT for factors such as procalcitonin (PCT), interleukin-6 (IL-6), and C-reactive protein (CRP) mostly includes compact chemiluminescence and fluorescence immunoassays. In 2024, a study showed that the Wondfo Finecare series had good precision in detecting PCT, with coefficients of variation (CVs) of 5 and 2.5% at concentrations of 0.5 ng/mL and 0.25 ng/mL, respectively (Mazlan et al., 2024). The Hotgen MQ60 and Minde CF10 use single-use packaging and chemiluminescence technology to detect more than a dozen different markers; through a combination of the high sensitivity of chemiluminescence with the flexibility of POCT, these systems have become the primary option for the dynamic monitoring of biomarkers of sepsis in emergency rooms and ICUs. New research in the field of POCT-based BSI detection and monitoring continues to produce interesting results. Chen C. H. et al. (2025) developed a rapid diagnostic system that combines lateral flow with a portable spectral reader to measure serum PCT and IL-6 levels, yielding semiquantitative results in approximately 30 min; these results were closely associated with those of traditional ELISA (Chen C. H. et al., 2025). Another study reported on a rapid dual-detection platform based on an electrochemical immunosensor in which 3D microgels encapsulating electroactive nanoprobes were used to measure the levels of PCT, IL-6, CRP, and pathogen marker lipopolysaccharide (LPS) in just 10 min from a 10 μL sample. The limits of detection were within pg./mL (IL-6: 3.4 pg./mL, PCT: 4.36 pg./mL, CRP: 5.9 pg./mL), significantly superior to those of conventional ELISA (Kaur and Singh, 2025).

While blood marker testing has become simpler and more convenient, molecular POCT platforms such as DiaSorin LIAISON PLEX and Gradientech QuickMIC are becoming more widely used for pathogen identification in clinical practice. LIAISON PLEX is a next-generation, FDA 510(k)-certified, molecular POCT platform that uses NanoGrid nonamplification chemistry to simultaneously detect 27 targets, including 19 gram-negative bacteria and 8 resistance genes (Diasorin, 2026). QuickMIC, developed by Gradientech in Sweden, has CE certification and an FDA breakthrough device designation. This system uses a microfluidic linear gradient for rapid antimicrobial susceptibility testing (AMT), and the corresponding clinical data have been verified (Berinson et al., 2024). Moreover, current cutting-edge research is focused on the greater integration of blood culture with testing to reduce the overall diagnostic time. The Qingdao Institute of Energy together with the Affiliated Hospital of Qingdao University developed a system based on static droplet array (SDA) chips that can directly analyze whole blood. The system completes quantitative detection, species identification, and phenotypic susceptibility testing for pathogenic bacteria in approximately 12 h (Diao et al., 2025). Moreover, a team in Taiwan developed PanAMR, a third-generation nanopore-based system that can identify 76 pathogens from positive blood cultures in 1 h and delivers accurate treatment recommendations in 15 h (Liu et al., 2024). These emerging technologies hold promise for achieving the ultimate goal of “sample-to-answer.”

### 3.3 Gastrointestinal infections

Gastrointestinal infections (especially community-acquired diarrhoea) are a major field for antibiotic abuse. In the aetiological diagnosis of viral diarrhoea, immunochromatography is the preferred tool in community clinics and primary medical institutions. Currently, the triple/quadruple test strips commonly used in clinical practice can detect pathogens such as rotavirus, norovirus, and adenovirus in 10–15 min. Compared with single- or double-pathogen detection kits, a triple immunochromatographic test kit that simultaneously detects three pathogens is more practical and convenient while maintaining comparable sensitivity and specificity (Ushijima et al., 2024). These strips are well suited for rapid triage in outpatient and emergency settings, but false negatives remain a concern. KUNPENG developed a five-pathogen nucleic acid test for infectious diarrhoea that uses RT–PCR to detect low viral loads with high sensitivity and specificity. Li et al. (2023) reported a single-tube, two-stage amplification method on the basis of reverse transcription recombinase-assisted PCR (RT-RAP) to simultaneously detect norovirus GII and group A rotavirus. The method delivers results within 1 h with a sensitivity of approximately 10 copies per reaction (Li et al., 2023).

For bacterial infections, POCT applications are more diverse. For example, colloidal gold testing is a common option alongside the urea breath test for detecting *Helicobacter pylori* (HP) (Best et al., 2018; Kubo et al., 2024; Silva Luz et al., 2025). In January 2026, an HP antigen test (colloidal gold method) developed by AIVIK Bio, a subsidiary of East Ocean, was approved by the National Medical Products Administration for the qualitative detection of HP antigens in stool samples (National Medical Products Administration, 2026). Regarding the development of new POCT platforms, Wang et al. (2025a) created a smartphone-based microelectrochemical immunosensor that can simultaneously detect anti-Ure antibodies to confirm infection and anti-CagA/VacA antibodies for strain typing, with a sensitivity of 94.12% and specificity of 93.33% relative to ELISA in 85 clinical samples.

Given the wide range of gastrointestinal pathogens, testing for a single pathogen at a time is inefficient, thus leading to growing interest in multiplex POCT (Szymczak et al., 2025a; Yoo et al., 2025; Henn et al., 2026). A recent study in Japan confirmed that FilmArray GI has excellent diagnostic performance in paediatric patients and that it can significantly outperform traditional methods in detecting viral gastroenteritis (Asagai et al., 2026). The QIAstat-Dx from Qiagen can detect 17 targets in 80 min, with positive rates greater than 90% and negative rates greater than 98.9% (Szymczak et al., 2025a), similar to the internationally validated FilmArray (Buss et al., 2015; Suleiman et al., 2024). Song developed a high-throughput centrifugal microfluidic system (MiLASD) that integrates nucleic acid purification, LAMP amplification, and real-time fluorescence signal analysis into a single CD microfluidic chip. This system can simultaneously detect 12 enteric pathogens (both bacterial and viral), with limits of detection of 10^2^ CFU/mL for bacteria and 10 copies/μL for viruses (Song et al., 2026).

### 3.4 Urinary tract infections

The most common POCT method for urinary tract infections (Sujith et al., 2024) remains the urine dipstick test, which achieves rapid screening through the detection of nitrites and leukocyte esterase. Its main advantages, including low costs, simplicity, and widespread availability, make it a primary tool for ruling out infections in emergency and outpatient settings. However, it cannot identify which pathogen is causing the infection or determine the corresponding AMR profile; moreover, it tends to miss nonnitratireductors such as enterococci and staphylococci, which makes guiding precision treatment difficult. The Micro Biological Survey (MBS) POCT platform also exhibits certain limitations (Arienzo et al., 2020); while it reduces the culture time to less than 5 h and accurately estimates the bacterial load (with 97% accuracy), it cannot identify the specific pathogen responsible.

To fill this gap, Joanna developed the Lodestar DX system, which uses LAMP to detect seven common urinary pathogens. A clinical validation study of this system involving 379 urine samples revealed an overall accuracy of 91.2% (Drazich-Taylor et al., 2025). Nevertheless, it does not detect resistance genes, making it inadequate for guiding appropriate antibiotic selection. The GeneXpert system offers the Xpert UTI test, which can detect multiple urinary pathogens and their resistance genes in approximately 50 min (Tops et al., 2023). Another option is the Vivalytic UTI analyzer, a PCR-based qualitative microarray platform. In a clinical evaluation of 126 patients, the system identified 19 bacterial species, achieving a diagnostic accuracy of ≥90.27% for 16 of them, while enabling the simultaneous detection of relevant resistance genes (Hartmann et al., 2024).

Advances in POCT for resistance gene detection are also worthy of mention. A team from the University of Giessen developed a custom LAMP panel. It can detect *Escherichia coli* (95.4% sensitivity, 96.1% specificity relative to conventional microbial culture) among 1,596 urine samples as well as six antibiotic resistance genes in 1 h, offering reliable support for antibiotic selection (Fritzenwanker et al., 2025). TubeCARE integrates urine processing, nucleic acid extraction, RPA amplification, and CRISPR/Cas12a detection in a sealed tube to provide automated “sample-to-answer.” The carbapenem resistance gene blaKPC was detected in 40 min, with a sensitivity of 1 CFU/mL (Yang et al., 2025). Newer CRISPR-based platforms such as BADLOCK combine RPA with CRISPR for identifying five common urinary pathogens and key resistance genes directly from urine samples in 1.5 h, with its pathogen identification accuracy reaching 98.8% (Roach et al., 2025). In addition, Yi reported a programmable, manually powered microfluidic (FPM) platform that supports biochemical and molecular analysis. The platform can run six urine biochemical tests in one minute and perform rapid phenotypic susceptibility testing in 3–5 h, with a diagnostic accuracy of up to 95.56% (Yi L. et al., 2025), bridging the gap from quick rule-out to precise treatment guidance. Notably, a bioinspired nanosensor offered a new approach to UTI testing. This sensor relies on the specific binding between *E. coli* FimH lectin and mannose, delivering visible results in 30 min without any equipment, with a detection limit as low as 10 CFU/mL. The validation results in 60 clinical samples (50 uropathogenic *Escherichia coli*-positive) suggest that this system could provide a cost-effective option for UTI screening in resource-limited areas (Priyadarshi et al., 2025).

### 3.5 Sexually transmitted infections and others

STIs, including HIV, syphilis, and hepatitis B, along with other priority diseases, such as tuberculosis, are key targets in the global public health field. Single-pathogen colloidal gold test strips for STIs serve as the main tools for early screening. On these bases, multiplex POCT strips now enable the screening of multiple STIs in a single test. A previous study from India indicated that multiplex strips are particularly suitable for self-testing among at-risk populations (Pai et al., 2012). GeneXpert is among the most widely used molecular POCT systems in clinical practice; it uses a modular design that allows different test cartridges to be interchanged to detect various pathogens. In addition to its well-known use for tuberculosis (Xpert MTB/RIF Ultra) (Acharya et al., 2020; Vaezipour et al., 2022), GeneXpert is also widely used for STIs. A multicenter study assessed the performance of GeneXpert in detecting *Chlamydia trachomatis* (CT) and *Neisseria gonorrhoeae* (NG) in men who have sex with men by using samples from both genital and extragenital sites. A total of 95.95% of the participants reported that they were willing to wait for results at the clinic, and staff reported that the system was stable and that the results were easy to interpret (Cordioli et al., 2024b). Another study involving high-risk women in four countries (Australia, Guatemala, Morocco, and South Africa) evaluated the ability of GeneXpert to detect CT, NG, and *Trichomonas vaginalis* (TV) and reported specificities above 96% relative to nucleic acid amplification technology among 1,383 patients (Shephard et al., 2024a). The GeneXpert platform is also applied for HIV testing. One study involving more than 1,200 HIV patients reported that the GeneXpert HIV-1 qualitative test had 99% sensitivity, specificity, and accuracy, while the quantitative performance was also above 90%. Qualitative assays were evaluated using a Roche CAP/C™, while Abbott m2000 was used as the reference system for quantitative detection (Bwana et al., 2019; Kamble et al., 2023).

Beyond GeneXpert, substantial progress has been made in other molecular point-of-care tests in the STI field. The Cobas Liat system (Roche) is another widely used platform; it uses real-time PCR and delivers results in 30 min. The CT/NG/MG assay can detect chlamydia, gonorrhoea, and *Mycoplasma genitalium* simultaneously (Hirsch et al., 2026). In 2025, Xie et al. (2025) and Hirsch et al. (2026) introduced the iPonatic system, which employs a one-step nucleic acid release technique, combines extraction, amplification, and detection in a disposable cartridge, and detects STI pathogens such as NG and CT within 30 min. The ProtonDx Dragonfly STI panel is designed to detect six pathogens, including gonorrhoea, chlamydia, and *Mycoplasma genitalium*, along with associated resistance genes. It has been preclinically validated and is scheduled for field evaluation in primary care settings. In March 2026, the Doherty Institute in Melbourne reported on a laboratory-developed, portable multiplex CRISPR-based detection prototype that can simultaneously test for syphilis, gonorrhoea, genital herpes, and chlamydia within 1 h (Low et al., 2026). Moreover, Rapidemic developed a 15-min portable molecular test that uses a disposable, device-free cartridge to detect CT and NG in just four steps (sample collection, lysis, amplification, and lateral flow readout) (Urazov et al., 2026).

In addition to identifying STIs, POCT methods have also been developed for detecting key infectious diseases, such as tuberculosis, malaria and viral hepatitis. The lateral flow urine lipoarabinomannan (LF-LAM) assay is a new, commercially available point-of-care test that detects lipoarabinomannan (LAM), a LPS present in mycobacterial cell walls, in individuals with active TB disease (Shah et al., 2016). Relative to microbiological testing as the reference standard, the new-generation PF-LAM (plasma fluorescence-enhanced LAM) test strip achieved a sensitivity of 58% (95%CI: 41–73%) and a specificity of 98% (95%CI: 88–100%) in diagnostic accuracy studies covering 399 well-characterized urine samples, outperforming the electrochemiluminescence LAM laboratory assay (Jiang et al., 2026). Another study offered a CLAP-TB (CRISPR/Cas-coupled RT-LAMP Amplification Protocol for Tuberculosis) assay, which can visually detect *Mycobacterium tuberculosis* from various clinical samples, such as sputum, urine and serum, within approximately 134 min (Kaushik et al., 2025). Although rapid diagnostic tests are easy to perform and inexpensive options for diagnosing malaria, they lack sufficient sensitivity. In controlled human malaria infection (CHMI) samples, LAMP was positive for all with parasitemia >1,000 parasites/ml, whereas RDTs were less reliable (59% positive for all with parasitemia >1,000 parasites/ml) (Payne et al., 2025). For the diagnosis of viral hepatitis, point-of-care HCV antigen (Ag) testing offers a promising alternative to RNA testing. Chen H. et al. (2025) developed a fully automated, smartphone-based POC HCV Ag assay using platinum nanoparticles, deep learning image processing, and microfluidics. A detection method for HCVcAg based on an electrochemical magnetic immunosensor demonstrated a limit of detection as low as 10 pg./mL, and its results were completely consistent with those of qPCR across 36 clinical plasma and serum samples (Abo-Zeid et al., 2025).

## 4 Discussion

### 4.1 Core functional concepts and scope of this review

POCT encompasses four core functions: pathogen detection, AMR gene detection, AST and microbial typing. Pathogen detection involves the qualitative or quantitative identification of target pathogens and their loads within clinical samples. AMR gene detection identifies genetic markers that mediate AMR at the molecular level. AST measures minimum inhibitory concentrations of different phenotypes to categorize isolates as susceptible, intermediate, or resistant. Microbial typing uses molecular fingerprints to resolve strain-level homology for epidemiologically investigating infectious disease outbreaks (Mahrous et al., 2026). However, numerous contemporary POCT publications frequently conflate these distinct functional categories under the umbrella term of “rapid diagnosis” when reporting the performance of POCT assays. Nonetheless, these four functions exhibit fundamental differences with respect to their importance in clinical decision-making, technical barriers to onsite implementation and the robustness of supporting clinical evidence. Pathogen detection helps determine the causative agent of infection, whereas AST informs optimal antimicrobial selection; however, AST requires a pure microbial culture and has therefore lagged substantially in the translation of point-of-care systems to clinical practice (Vasala et al., 2020).

By covering technologies ranging from LFIAs to fully integrated molecular diagnostic systems, we specifically discuss evidence-driven technology selection in different clinical contexts (outpatient screening, emergency-department decision-making, intensive-care surveillance, and epidemic outbreak response), wherein prioritization balances assay turnaround speed and analytical sensitivity rather than reflecting a simplistic ranking based on technological sophistication (Tables 2, 3). Furthermore, resistance gene detection and phenotypic AST are framed as key analytical components instead of secondary analyses for pathogen identification. This perspective arises from the strict timeliness constraints imposed by AMR management. The clinical utility of resistance profiling is markedly reduced when results are unavailable within the therapeutic decision-making window for timely antimicrobial administration (Mahrous et al., 2026). Finally, implementation-relevant parameters for resource-limited settings, including cost-effectiveness, workforce training, quality assurance, and supply-chain stability, are discussed for each technology. The ability of POCT to mitigate the global infectious disease burden relies on its sustainable deployment in low-resource settings rather than its satisfactory performance exclusively within well-resourced tertiary care centers (Drain et al., 2014; Shaetonhodi et al., 2026).

**Table 2:** Comparison of major POCT technology platforms for infectious disease diagnosis.

Technology platform	Representative system	Sensitivity	Specificity	Turnaround time	Cost	Equipment requirements	Applicable function type	Key advantages	Key limitations
LFIA	HealgenCOVID-19/Influenza A/B Combo Rapid Test	Highly dependent on viral load; sensitivity decreases significantly at low viral loads	High (>95%)	10–15 min	Very low	None	Pathogen detection, (qualitative), typing (limited, serotype-specific)	Rapid, user-friendly, inexpensive, suitable for home use	Qualitative/semi-quantitative, sensitivity drops at low viral loads, window-period false negatives
CLIA	HotgenMQ60/Mindray CF10	At the pg./mL level	High	15–30 min	Medium	Benchtop instrument	Pathogen detection (quantitative)	Quantitative, broad linear range, suitable for dynamic monitoring	Benchtop size, higher cost per test than a LFIA, limited to biomarker detection
IAT	Lodestar DX/G4-NASBA	LOD: 1–3 copies/μL (single-copy level)	High (primer-dependent)	15–60 min	Medium	Portable heating device	Pathogen detection, resistance gene detection, typing (research stage)	High sensitivity, no thermal cycling needed, portable	Complex primer design, risk of non-specific amplification, quantitative capability varies
Multiplex PCR	BioFire SPOTFIRE/GeneXpert	Agreement with reference: >94%	High	15–90 min	Medium–high	Dedicated analyzer	Pathogen detection, resistance gene detection, typing	High multiplexing capability, reliable results, widely clinically validated	High equipment cost, requires trained operators, laboratory-like environment preferred
CRISPR/Cas	SHERLOCK/DETECTR/BADLOCK	At the aM level (with pre-amplification)	Very high	1–1.5 h	Low–medium	Isothermal device or fluorescence reader	Pathogen detection, resistance gene detection, typing (research stage, SNP-based)	Ultra-high specificity, flexible assay design, high multiplex potential	SHERLOCK/DETECTR have been validated in multiple studies but are not yet suitable for clinical practice, BADLOCK is at early validation stage (preprint)
Fully integrated microfluidics	BioFire FilmArray (clinical use)/MiLASD or SDA chip (research stage)	Comparable to reference methods	Comparable to reference methods	1–2 h	High	Dedicated cartridge-based reader	Comprehensive (pathogen + resistance, AST in research, typing in research)	Fully automated, “sample-to-result”, reduced contamination risk, minimal operator dependence	Manufacturing scalability challenges, high costs, few commercially successful products
Rapid AST systems	QuickMIC/FPM platform (research stage)	Agreement with broth microdilution: >95%	High	2–5 h	High	Specialized microfluidic instrument	Antimicrobial susceptibility testing (AST)	Direct MIC measurement, can guide precision antibiotic therapy selection	Requires positive culture, technically demanding, very few products commercially available

LFIA, lateral flow immunoassay; CLIA, chemiluminescence immunoassay; IAT, isothermal amplification technology; PCR, polymerase chain reaction; CRISPR, clustered regularly interspaced short palindromic repeats; LOD, limit of detection; MIC, minimum inhibitory concentration; AST, antimicrobial susceptibility testing; MLST, multilocus sequence typing; SNP, single-nucleotide polymorphism; PPA, positive percent agreement; NPA, negative percent agreement; ESBL, extended-spectrum beta-lactamase; CE, Conformité Européenne; FDA, U.S. Food and Drug Administration. Applicable function type indicates the primary clinical question each platform can address: pathogen detection (identifying the pathogen responsible), resistance gene detection (identifying genetic resistance markers), AST (determining which antibiotic will work), and microbial typing (tracing whether the pathogen is responsible for an outbreak). Platforms annotated with “research stage” or “preprint” have not yet received regulatory approval for routine clinical use.

**Table 3:** Overview of POCT technology platforms for infectious diseases.

Infection type	Technology platform	Principle	Time	Sensitivity/ Accuracy	Quantification	Cost	Main limitations
Respiratory tract infections	Immunochromatography strip (Sohrabi et al., 2022) (Healgen)	Colloidal gold	5–15 min	>77% at high viral load, 32–77% at moderate load, 7% at low load (Nguyen Van et al., 2025)	Qualitative	Very low	Missed detection at low viral loads
	Coyote Flash10 (Smit et al., 2025)	Fast PCR	30–36 min	94.6% agreement with GeneXpert (Smit et al., 2025)	Qualitative	Medium	Requires instruments
	G4-NASBA (Luo et al., 2026)	NASBA isothermal	15 min	LOD 3 copies/μL	Qualitative	Medium	Research stage
	BioFire SPOTFIRE (Meltzer et al., 2026)	Multiplex PCR	15 min	98.5% PPA, 99.1% NPA	Qualitative	High	High equipment cost
	Microfluidic biosensor (Ramirez-Priego et al., 2026)	Plasmonic resonance	Not specified	Sensitivity 97.22%, specificity 100%	Quantitative	Medium-high	Research stage
Bloodstream infections	Hotgen MQ60/Minde CF10	Chemiluminescence	15–30 min	pg/mL level	Quantitative	Medium	Biomarkers only
	LIAISON PLEX (Diasorin, 2026)	Non-amplification NanoGrid	2 h	LOD 7.77 × 10^2^ CFU/mL	Qualitative	High	Requires positive blood cultures
	QuickMIC (Berinson et al., 2024)	Microfluidic gradient	2–5 h	>95% agreement	MIC-quantitative	High	Requires positive blood cultures
	SDA chip system (Diao et al., 2025)	Microdroplet culture	12 h	LOD 10 CFU/mL	MIC-quantitative	Medium	Research stage
	PanAMR (Liu et al., 2024)	Nanopore sequencing	1–15 h	>90% agreement	Qualitative	Medium-high	Requires positive blood cultures
Gastrointestinal infections	Triplex/quadruplex immunochromatography (Ushijima et al., 2024)	Colloidal gold	10–15 min	100% specificity	Qualitative	Very low	Missed viral detection
	RT-RAP single-tube amplification (Li et al., 2023)	Recombinase-assisted PCR	1 h	Sensitivity ~10 copies/reaction	Qualitative	Medium	Research stage
	MiLASD microfluidic system (Song et al., 2026)	LAMP	45 min	Bacteria: 10^2^ CFU/mL, virus: 10 copies/μL	Qualitative	Medium	Research stage
	FilmArray GI Panel (Yoo et al., 2025)	Multiplex PCR	1 h	sensitivity 98.5%, specificity 99.2%	Qualitative	High	High equipment cost
	QIAstat-Dx (Szymczak et al., 2025a)	RT–PCR	1 h	sensitivity 90%, specificity 99%	Qualitative	High	High equipment cost
Urinary tract infections	Urine dipstick (Sujith et al., 2024)	Chemical colorimetry	1–2 min	sensitivity of nitrite 23.31%, leukocyte esterase 48.5%, and blood 63.94%	Semi-quantitative	Very low	No pathogen identification
	MBS POCT (Arienzo et al., 2020)	Rapid culture	5 h	Bacterial load accuracy 97%	Quantitative	Medium	No species identification
	Lodestar DX (Drazich-Taylor et al., 2025)	LAMP	35 min	Overall accuracy 91.2%, *E. coli* sensitivity 86%, specificity 88%	Qualitative	Medium	No resistance detection
	GeneXpert UTI (Tops et al., 2023)	Real-time PCR	50 min	ESBL detection sensitivity 90.4%, specificity 98.4%	Qualitative	Medium-high	Higher equipment cost
	Custom LAMP panel (Fritzenwanker et al., 2025)	LAMP	1 h	*E. coli* sensitivity 95.4%, specificity 96.1%	Semi-quantitative	Medium	Only for *E. coli*
	TubeCARE (Yang et al., 2025)	RPA + CRISPR	40 min	blaKPC LOD 1 CFU/mL	Qualitative	Low-medium	Single target
	BADLOCK (Roach et al., 2025)	RPA + CRISPR	1.5 h	Pathogen ID accuracy 98.8%	Qualitative	Low-medium	Preprint
	FPM platform (Yi L. et al., 2025)	Microfluidics + biochemistry	1 min + 3–5 h	AST accuracy 95.56%	MIC-quantitative	Low-medium	Research stage
	Gold nanoparticle colorimetric sensor (Priyadarshi et al., 2025)	Glycan-lectin binding	30 min	LOD 10 CFU/mL	Semi-quantitative	Very low	Only for *E. coli*
Sexually transmitted infections	Multiplex immunochromatography (Pai et al., 2012)	Colloidal gold	10–20 min	Syphilis TP antibody sensitivity 90.1%, specificity 97.9%	Qualitative	Very low	Missed detection during certain windows
	GeneXpert CT/NG/TV (Cordioli et al., 2024b)	Real-time PCR	~90 min	CT/NG sensitivity 84–91%, specificity >99%	Qualitative	Medium-high	High equipment cost
	GeneXpert HIV (Bwana et al., 2019; Kamble et al., 2023)	Real-time PCR	~90 min	Qualitative: sensitivity, specificity, accuracy 99%	Quantitative	Medium-high	High equipment cost
	Cobas Liat (Hirsch et al., 2026)	Real-time PCR	20–30 min	sensitivity 92%, specificity 97%	Qualitative	Medium-high	High equipment cost
	iPonatic (Xie et al., 2025)	Rapid nucleic acid release	30 min	AUC 98%, sensitivity 95%, specificity 100%	Qualitative	Medium	Pre-clinical
	Multiplex CRISPR platform (Low et al., 2026)	CRISPR/Cas	1 h	Sensitivity T pallidum 82.5%, pan-HSV 94.4%	Quantitative	Low-medium	Research stage
	Rapidemic molecular test (Urazov et al., 2026)	Isothermal + LFD	15 min	100% agreement with gold standard for CT/NG	Qualitative	Very low	Pre-clinical

LAMP, loop-mediated isothermal amplification; RPA, recombinase polymerase amplification; NASBA, nucleic acid sequence-based amplification; CRISPR, clustered regularly interspaced short palindromic repeats; RT–PCR, reverse transcription polymerase chain reaction; LOD, limit of detection; MIC, minimum inhibitory concentration; PPA, positive percentage agreement; NPA, negative percentage agreement; ESBL, extended-spectrum beta-lactamase; CT, *Chlamydia trachomatis*; NG, *Neisseria gonorrhoeae*; TV, *Trichomonas vaginalis*; CFU, colony-forming unit. Quantification categories: “Qualitative” indicates a yes/no result; “semi-quantitative” indicates an approximate level (e.g., trace, moderate, strong); “quantitative” indicates a numeric result; “MIC-quantitative” indicates a numeric minimum inhibitory concentration value. Main limitations: “Research stage” indicates that the platform has been validated in laboratory studies but has not yet entered routine clinical use; “Preclinical” indicates that the platform has been validated in vitro or in animal models but not in human clinical trials; and “Preprint” indicates that the corresponding study has not yet been peer reviewed.

### 4.2 Technology spectrum and selection logic

Elucidating functional hierarchies will facilitate a more nuanced appreciation of the differences among diverse POCT platforms. In recent years, POCT methods for infectious diseases have undergone rapid development (Prakash et al., 2026), ranging from simple immunochromatographic test strips to highly integrated molecular detection systems, deployed across various platforms. These technologies do not replace each other; rather, they form a continuous spectrum, reflecting the fundamental trade-offs between analytical performance and ease of use (Figure 1). At one end of the spectrum, the immunochromatographic assay is extremely fast, inexpensive, and uses no other equipment, making it highly suitable for large-scale screening and home self-testing. However, it can provide only qualitative results and has low sensitivity. On the other end, fully integrated “sample-to-answer” platforms can deliver sensitivity and specificity close to those achieved in the laboratory in 1–2 h at the expense of significantly higher costs and more complex operation (Bai et al., 2025; Bi et al., 2025). Among these methods, isothermal amplification methods (such as LAMP and RPA), as well as compact multiplex PCR or chemiluminescence systems, provide balanced solutions that optimize testing speed, sensitivity, and multiplexing capability for primary care and emergency settings.

**Figure 1:**
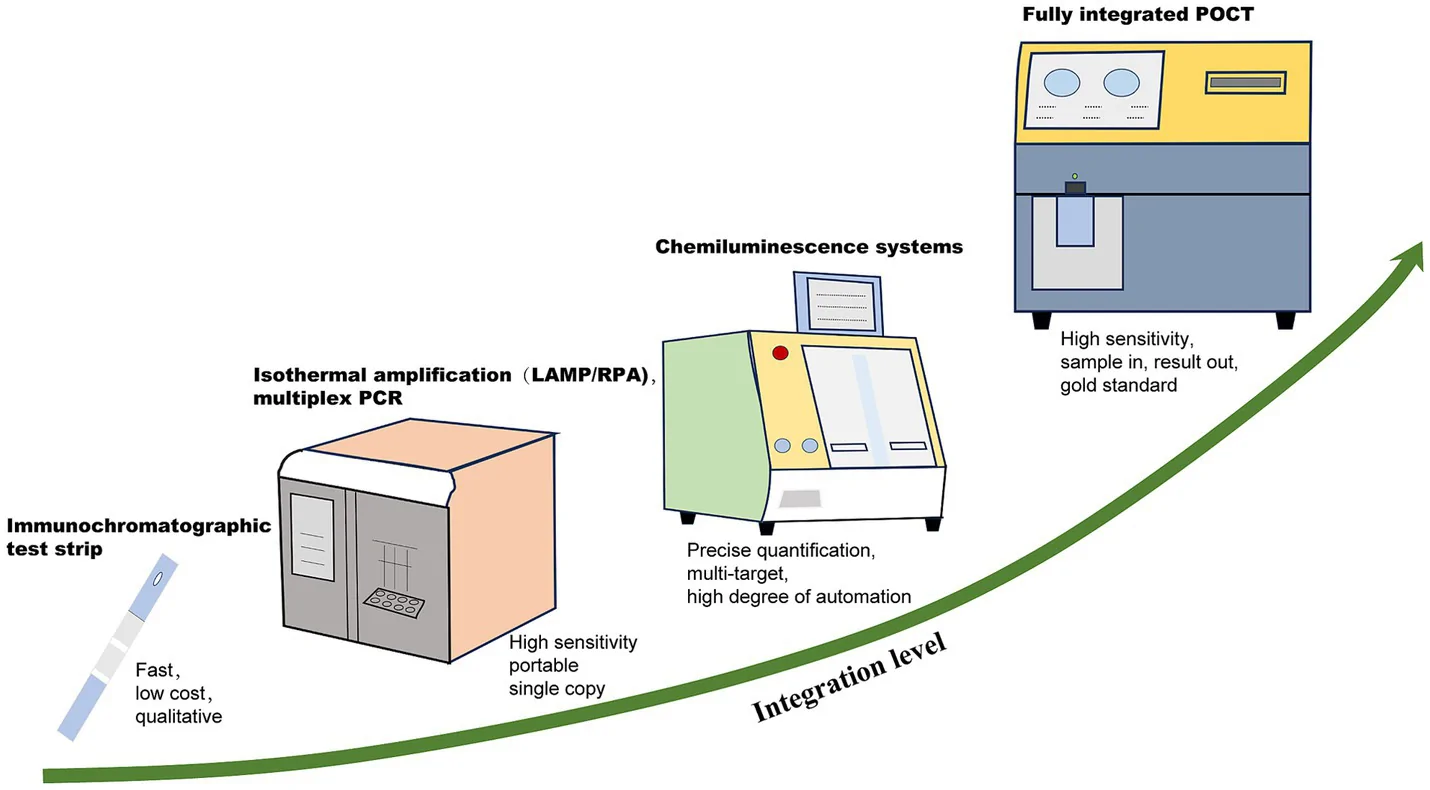
Technology spectrum of infectious disease point-of-care testing technologies. This figure illustrates four representative forms of POCT. The green curve denotes progressive increases in integration level and cost and does not represent a performance-based ranking of the products. Real-world technology selection should be considered in terms of clinical requirements (assay speed, analytical sensitivity, sample throughput) and on-site resource accessibility. From left to right: (1) Immunochromatographic test strips (fast, low-cost, qualitative for large-scale screening). (2) Isothermal amplification (LAMP/RPA) and multiplex PCR platforms (portable, single-copy sensitivity for point-of-care nucleic acid testing). (3) Chemiluminescence systems (precise quantification, multitarget detection, and high automation for ED/ICU diagnosis). (4) Fully integrated POCT systems (“sample-to-answer” gold-standard performance for critical care but currently expensive and low throughput).

### 4.3 Implementation challenges and stewardship perspectives

While POCT has undergone substantial development and shows great promise for infectious disease diagnosis, it also faces multiple challenges in its translation from the laboratory to the clinic and in evolving from a supporting tool to a central diagnostic modality. One of the relevant issues that remains to be sufficiently addressed is sensitivity. A study from South Africa on COVID-19 antigen POCT demonstrated that while the sensitivity and specificity were above 77% in samples with high viral loads (Ct < 25), they decreased sharply to 32% in samples with moderate viral loads (Ct 25–35) and just 7% in those with low viral loads (Ct > 35) (Rukasha, 2025). This means that such antigen tests are likely to miss cases during early infection, when viral loads are low. These false negatives not only delay treatment but also facilitate disease spread because of the misjudgment of the patient’s condition.

Another critical limitation relates to quantitation. Most conventional immunochromatography tests provide only qualitative results, which can substantially limit attempts to manage chronic conditions or track treatment responses. For example, PCT levels can help guide the initiation or discontinuation of antibiotics (Schuetz et al., 2017; Kiya et al., 2024), while the viral load of hepatitis B needs to be measured accurately to evaluate whether antiviral therapy is effective (Choi et al., 2024; Gu et al., 2024). Molecular POCT platforms (isothermal amplification or PCR-based systems) offer high sensitivity and yield quantitative results, but they are expensive and highly complex (Augustine et al., 2020; Bruijns et al., 2022). This complexity is associated with another challenge: quality control. POCT is intended for use by nonspecialized personnel, but this practicality is often compromised when the tests involve sophisticated molecular diagnostic devices. Variability in sample collection, reagent measurement and readout time can affect the results. Furthermore, the wide diversity of POCT brands and technologies on the market can contribute to inconsistent results across different devices and even batches, which hinders the ability of clinicians to interpret results with confidence and limits data integration and comparability across studies.

In low-resource areas, the challenges extend beyond technological ones; cost and management are equally critical factors. The real-world translation of POCT into tangible clinical and public health benefits requires its implementation strategies and accompanying governance frameworks. Experience suggests that the implementation of reasonable reimbursement policies and exploration of equipment-sharing models may help make POCT more affordable and sustainable (Vogler et al., 2024). Once a test is performed, however, the subsequent steps are equally critical: as revealed in a recent study, the successful implementation of POCT also requires a clear “diagnosis–treatment–referral” pathway (Nguyen Van et al., 2025). For example, when a reactive rapid HIV test is performed, robust mechanisms must be in place to ensure timely confirmatory testing and prompt initiation of antiretroviral therapy. Similarly, in patients diagnosed with syphilis, standardized protocols should be implemented to notify sexual partners and conduct effective contact tracing.

Community–based pharmacies are the most promising non-traditional settings for expanding POCT. A Canadian survey of 4,035 community pharmacists reported that 72.7% of respondents offered POCT services, with pharmacist-led testing for Group A streptococcal pharyngitis effectively reducing inappropriate antibiotic prescription (Abdellatife et al., 2026). Nevertheless, large-scale implementation is limited by notable systemic barriers, including ambiguous regulatory mandates, a lack of standardized training, inadequate referral pathways linking pharmacies to primary-care facilities, and incomplete reimbursement frameworks. A survey conducted in the United Arab Emirates revealed that 66.4% of community pharmacists regarded standardized protocols for communicating the results of POCT as an urgent unmet need (Jarab et al., 2025). In low- and middle-income countries, weak regulatory governance and volatile supply chains further compound these obstacles (Jarab et al., 2024). The integration of POCT into the clinical workflow also poses considerable challenges within primary care settings. A qualitative evaluation from the United Kingdom revealed that clinicians frequently resort to batch testing instead of POCT owing to time constraints, which substantially undercuts the core merits of POCT (Xie et al., 2024). Findings from Germany further illustrated that although CRP POCT facilitates discrimination between viral and bacterial infections, the associated increases in hands-on testing and patient consultation time, together with insufficient statutory reimbursement, are major practical barriers to its implementation in primary care (Jung et al., 2025). Institutional support, early-stage clinician engagement, structured training programs, and the integration of POCT into antimicrobial stewardship teams are core strategies for mitigating such barriers (Nguyen Van et al., 2025). These implementation barriers for POCT are most pronounced in resource-limited contexts. However, systematic reviews have identified rapid turnaround times, portability, and user-friendliness as enabling factors, whereas weak supply chains, unsustainable funding streams, training gaps, and workforce shortages are key limitations (Chaudhary et al., 2025; Shaetonhodi et al., 2026). In AMR-related diagnostics, access to testing services has been shown to be markedly inequitable between urban and rural populations (Modgil et al., 2025). Lessons from COVID-19 antigen testing indicate that even this relatively simple diagnostic platform is associated with systemic hurdles related to operational protocols, result reporting, and quality oversight during real-world deployment in low-income nations (Mangenah et al., 2025).

### 4.4 Evidence quality, gaps, and limitations

The present review has several inherent limitations. As this was a narrative review, a quantitative meta-analysis was not performed. Consequently, the performance metrics, including sensitivity and specificity, cited herein stem from the qualitative synthesis of heterogeneous study findings. Accordingly, between-study variability in sample sizes, reference standards, and experimental design may have been underappreciated, and thus, caution is warranted when attempting to generalize the findings from single studies to overall platform performance. Second, although most evaluations of POCT systems employed a cross-sectional, diagnostic–accuracy design (Bossuyt et al., 2015), only a minority of studies have provided rationales for their sample size calculation (Kirby et al., 2024; Beaubien-Souligny et al., 2025). Several studies have enrolled only 6–20 participants, which is insufficient for validating the real-world clinical applicability of diagnostic assays. With respect to reference-standard selection, most studies have adopted laboratory-based RT–PCR or microbial culture. However, reporting on the management of discordant samples was frequently insufficiently transparent in terms of whether an independent third-party assay was performed for validating the results. In terms of head-to-head comparisons, while a subset of studies directly benchmarked the performance of different POCT platforms, such studies are relatively uncommon (Smit et al., 2025; Kavunga-Membo et al., 2026). Moreover, most reports compared novel diagnostic technologies solely against a single gold standard without assessments against competing POCT modalities. This gap leaves clinical decision-makers without direct comparative evidence when multiple alternatives to commercial assays are available.

Critical evidence gaps also persist within the POCT landscape. Across infection types, the evidentiary quality of POCT studies is uneven. Respiratory infections and STIs have the largest bodies of clinical validation data, which are backed by multicenter trials and real-world assessments (Cordioli et al., 2024a; Shephard et al., 2024b; Meltzer et al., 2026; Ramirez-Priego et al., 2026). Even in these well-investigated fields, however, most diagnostic-performance data originate from tertiary-care centers testing biobanked specimens; this limits how well the findings translate to primary-care clinics, community pharmacies, and home-testing contexts. For BSIs, the findings concerning host biomarkers, including PCT and IL-6, are supported by solid evidence. In contrast, molecular platforms built to directly identify pathogens and detect resistance are still in the early stages of development, and the vast majority still require positive blood culture results, negating much of their potential to reduce diagnostic turnaround time (Berinson et al., 2024). In gastrointestinal and urinary tract infections, molecular panels such as FilmArray GI, QIAstat-Dx, and GeneXpert UTI deliver good diagnostic accuracy but have notable trade-offs in terms of cost and hardware requirements. Importantly, clinicians rely on genotypic resistance markers to guide treatment decision-making, but the clinical link between these genotypic markers and phenotypic susceptibility remains poorly defined (Drazich-Taylor et al., 2025; Szymczak et al., 2025b). For tuberculosis and malaria, LF-LAM and rapid diagnostic tests (RDTs) have known sensitivity gaps in certain patient subgroups, such as immunocompetent individuals or those with low parasitaemia, limiting their practical value in real-world settings (Jiang et al., 2026).

From a technical standpoint, most published reports on CRISPR/Cas diagnostics, acoustofluidic chips and selected advanced microfluidic platforms remain limited to proof-of-concept or small-scale validation studies. Prospective, multicenter, large-sample real-world clinical studies are lacking, and the consistency of their diagnostic performance under suboptimal field conditions remains poorly understood (Wang et al., 2025b; Tanifuji and Citterio, 2026). Geographically, the included literature is heavily skewed towards studies published in North America, Europe, and East Asia, with a notable underrepresentation of investigations from high-burden infectious-disease regions, including South Asia, Africa, and Latin America (Drain et al., 2014; Shaetonhodi et al., 2026). This imbalance not only limits the generalizability of the conclusions synthesized in this review but also indicates that ongoing POCT studies objectively prioritize clinical demands specific to high-income settings. At the translational level, the majority of studies focus on analytical performance metrics, while real-world implementation data addressing cost-effectiveness, operator adherence, and tangible impacts on clinical decision-making and antimicrobial prescribing remain exceedingly scarce (Mahrous et al., 2026; Nguyen Van et al., 2025). These gaps represent key priorities for future research: conducting multicenter prospective head-to-head validation studies, integrating health-economic evaluations and implementation-science approaches into standard POCT research frameworks, and scaling up field-validation initiatives in low- and middle-income countries.

### 4.5 Future directions

Currently, POCT is transitioning from a support tool to a highly central element in diagnostics. The development of new materials and methods continues to expand the limits of sensitivity: nanomaterials can improve signal strength by several orders of magnitude, while CRISPR technology has introduced a new level of specificity for nucleic acid detection. In May 2025, Southeast University and Getein Biotech reported a prototype handheld molecular POCT system that uses RPA and microfluidics to test for eight respiratory pathogens in 25 min, with a sensitivity above 97% and a specificity over 99% (Zai et al., 2025). Moreover, “sample-to-answer” is becoming the new standard for top-end POCT. Fully integrated systems that combine microfluidics and automation require the users only to load a sample and press start, after which the test is performed in a sealed, self-contained cartridge. Digital tools implement components such as artificial intelligence that could help read results more accurately, while integration with 5G and the IoT would allow the results to be transmitted remotely and analysed in the cloud (Chen et al., 2024). A more connected, digital framework for infectious disease control should therefore be constructed (Wei et al., 2025; Li et al., 2026b).

In the future, POCT is likely to evolve into a robust and independent element of the diagnostic landscape rather than just a supplementary adjunct to central labs. To this end, technical standards should be developed to ensure that the results from different devices and batches can be compared and trusted. Quality control systems also need to be adapted and optimized for nontraditional testing environments. Implementation of the standards of ISO/TS 22583:2019(E) and GB/Z 43281(2023) represents a critical step towards this goal (ISO, 2019; NIFDC, 2024). Moreover, clinical application should be standardized to ensure that the integration of POCT with conventional laboratory testing establishes a new model characterized by a clear division of labor, coordinated collaboration, and complementarity advantages between the two approaches. Whether deployed in emergency rooms, community clinics, patient homes, or remote villages, POCT systems have the potential to become a true part of the healthcare system and meaningfully improve the management of infectious diseases worldwide.
